# Familial high-risk (FHR) for severe mental illness in clinical high-risk for psychosis (CHR-P): a systematic review and meta-analysis

**DOI:** 10.1007/s00406-026-02267-1

**Published:** 2026-05-28

**Authors:** Andrea Raballo, Michele Poletti, Antonio Preti

**Affiliations:** 1 REMEDI Lab Euler Institute and Chair of Psychiatry, Faculty of Biomedical Sciences, University of Southern Switzerland, Lugano, Switzerland; 2 Cantonal Sociopsychiatric Organisation, Mendrisio, Switzerland; 3 Department of Mental Health and Pathological Addiction, Child and Adolescent Neuropsychiatry Service, Azienda Unità Sanitaria Locale-IRCCS di Reggio Emilia, Reggio Emilia, Italy; 4 Department of Neuroscience, University of Turin, Turin, Italy

**Keywords:** Familial high-risk, Severe mental illness, Genetic risk, Clinical High-Risk for Psychosis, First and second-degree offspring, Transition to psychosis

## Abstract

Preventive interventions for young help-seekers at clinical high risk for psychosis (CHR-P) critically depend on the progressive improvement of risk stratification. However, there is evidence that the transparent reporting of relevant variables plausibly affecting longitudinal outcomes (e.g. pre-assessment pharmacological exposure) remains suboptimal. In this study we focused on familial high-risk (FHR) for severe mental illness in CHR-P studies, as regards reporting habits, prevalence and possible role in the transition to psychosis. We performed a systematic review and meta-analysis on studies published on Medline and the Cochrane Library on CHR-P individuals with possible transition as outcome, and published from inception to March 31, 2024. The literature review identified 93 independent samples. Up to 26% of studies included information on first-degree relatives with psychosis. The estimated proportions of FHR for severe mental illness were 29.9% (95% CI: 28.2%–31.6%) for a broad family history of psychiatric illness, 21.0% (95% CI: 19.7%–22.4%) for a family history of psychosis in first-degree relatives, and 24.6% (95% CI: 21.7%–27.7%) for a family history of psychosis in either first- or second-degree relatives. In the small subgroup of studies reporting this information, a family history of psychosis in a first- or second-degree relative was negatively associated with the risk of transition to psychosis. No other significant associations were found between features of familial high risk (FHR) and transition to psychosis. Overall, FHR for severe mental illness is poorly reported in observational cohorts of CHR-P individuals, although it should be *ordinarily* screened during assessment according to validated instruments. Several limitations may account for the current lack of evidence, highlighting the need for further empirical investigation.

**Supplementary Information:**

The online version contains supplementary material available at https://doi.org/10.1007/s00406-026-02267-1.

## Introduction

Despite the progressive refinement of prognostic calculators [1, 2] and the widespread aspiration toward precision psychiatry [3], the field of Clinical High-Risk for Psychosis (CHR-P) still suffers from endemic, largely undebated conceptual shortcuts. For example, the conceptualization and detailed reporting of relevant variables that may impact longitudinal outcomes remain suboptimal [4, 5]. One such variable is baseline exposure to pharmacological treatments [6], which can act as a potential confounder for clinical presentation at enrollment and as a possible modulator of longitudinal outcomes [7–11].

Another key variable to improve prognostic precision, which is only marginally considered in its full potential, is Familial High-Risk (FHR) for severe mental illness. Multiple empirical methodologies - from classic designs such as twin studies [12–14] and FHR studies on offspring [15–17] to more recent polygenic risk scores derived from genome-wide association studies [18] - have consistently confirmed the high heritability of severe mental illnesses such as schizophrenia, bipolar disorder, and major depression, as well as the broader transdiagnostic risk for mental illness in offspring of patients with severe mental illness. Furthermore, within the broad area of heritability, there is a progressive increase in risk according to the degree of genetic relatedness - i.e., a higher risk in first-degree relatives compared to second-degree relatives [19].

The importance of FHR for severe mental illness was recognized in the original conceptualization of the early detection approach [20, 21], which included the Genetic Risk and Deterioration Syndrome (GRDS) criteria alongside other CHR-P criteria, namely Attenuated Psychotic Symptoms (APS) and Brief Limited/Intermittent Psychotic Symptoms (BLIPS). Specifically, GRDS is defined by a combination of presumed genetic risk (i.e., family history of psychosis or individual diagnosis of schizotypal personality disorder) and functional decline or sustained low functioning. However, GRDS appears to be associated with lower transition rates to psychosis (compared to APS and BLIPS) and has a low meta-analytical prevalence (5% among CHR-P samples) [22].

This finding is somewhat surprising given that CHR-P represents a clinically enriched high-risk population for psychosis, and severe mental disorders such as affective and non-affective psychoses are highly heritable. This incongruence might stem from the fact that GRDS only captures a fraction of the broad spectrum of FHR - specifically, individuals with concomitant functional decline - while its proper classification may be overshadowed by more overt presentations, such as APS or BLIPS. This issue could have several non-trivial consequences.

First, FHR for severe mental illness in CHR-P cohorts is likely underestimated. Second, heritability and FHR remain relatively marginal (if not largely ignored) as outcome predictors in existing prognostic models (i.e., risk calculators) of transition to psychosis in CHR-P cohorts. Third, even when FHR is reported, there may be inconsistencies in how it is defined - for example, considering only the presence of a first-degree relative with psychosis (i.e., FHR in the narrow sense) or including a broader definition that encompasses any psychiatric diagnosis in first- and second-degree relatives (i.e., FHR in the broad sense).

Therefore, the specific aims of this study are to:

- *conduct a systematic review of reporting practices regarding FHR for severe mental illness in observational cohorts of CHR-P individuals reporting transition outcomes;*
- *perform a meta-analysis of the prevalence of FHR for severe mental illness in observational cohorts of CHR-P individuals reporting transition outcomes;*
- *assess whether FHR influences longitudinally the likelihood of transition to psychosis.*

## Methods

### Data source and search strategy

This study has been set up according to the suggestions of the Preferred Reporting Items for Systematic Reviews and Meta-Analyses (PRISMA) [23] and its revision [24]. The following search engines were probed: PubMed/Medline (https://pubmed.ncbi.nlm.nih.gov/) and the Cochrane Library (https://www.cochranelibrary.com/). The time interval was from inception up to March 31, 2024. A combination of the following key terms was applied: ‘Ultra high risk’ OR ‘Clinical high risk’ AND ‘psychosis’ AND ‘transition’ OR ‘conversion’.

The results of the search were inspected by two investigators with minimum ten years of experience *in conducting systematic reviews* and cross-checked by a third investigator expert in the field. The following criteria were applied to retain an article in the study: the article was written in English; it included samples with people diagnosed CHR-P based on a validated diagnostic procedure; it reported numeric data about the sample and the outcome at a predefined follow-up time; it had transition to psychosis, as defined according to pre-specified criteria, as one of the outcomes.

## Data extraction

Independent studies were included when they matched the inclusion criteria. Articles that were unrelated to the main topic (e.g., studies on brain imaging or genetic markers) and duplicates (e.g., articles repeatedly reporting the results of the same trial with overlapping samples) were excluded, retaining those more detailed and informative about FHR.

When discrepancies did arise in judging about correspondence of the article to the inclusion criteria or about considering it a duplicate publication, a discussion with a third experienced researcher was used to take a final decision. All references of the scanned articles and of the available reviews on the topic were looked for potentially missed studies.

At the end of this procedure, 93 independent studies were included in the systematic review (PRISMA flow chart: Figure S1 in Supplementary materials).

Two experienced investigators extracted the following variables on the basis of a predefined protocol: authors and year of publication of the study; location of the study; criteria and instrument for diagnosis; criteria for transition to psychosis; sample size at baseline and at follow-up; mean age in the sample; gender ratio in the sample; duration of the follow-up, FHR for severe mental illness. In the studies, FHR for severe mental illness has been described both as a broad occurrence of a mental disorder among biological relatives and specifically about psychosis within the spectrum of schizophrenia. Some studies reported a FHR for psychosis among first-degree relatives (essentially, parents or siblings). Some studies also reported information about a FHR for psychosis among second-degree relatives, i.e., uncles, aunts, nephews, nieces, grandparents, grandchildren, and cousins. We therefore extracted and described information about: broad FHR for severe mental illness; FHR for psychosis among first-degree relatives; FHR for psychosis among second-degree relatives.

### Statistical analysis

The analysis was done with the IBM SPSS Statistics for Windows, Version 27.0, and with the ‘meta’ package [25] and the ‘metafor’ package [26] running in R version 4.3.2 (R Core Team, 2023). Alpha was set at *p* < 0.05, and we reported both point estimations and 95% confidence interval (CI).

A random intercept logistic regression model – also known as logit transformation - was used to estimate all proportions [27] since the variance-stabilizing Freeman and Tukey double arcsine transformation [28], a preferred method in the meta-analysis of proportions, might lead to misleading results in a meta-analysis of single proportions due to problems with the back-transformation of the Freeman-Tukey transformation [29].

Between- and within-studies variance were estimated with the τ^2^ statistics using Empirical Bayes estimator [30] and its 95% CI, which was calculated by using the Q-Profile method [31] with Knapp and Hartung correction [32]. Continuity correction of 0.5 was applied in studies with zero cell frequencies. For all analyses, we reported the results of both the fixed-effects and the random-effects model. However, in the interpretation of the results, we gave prominence to the fixed-effects model. Indeed, we were interested in the actual prevalence in the reviewed studies and were not aimed at producing an estimate of the prevalence of FHR in the population from which the studies were drawn. At the moment, this population is unknown because of the scarce reporting of data. Heterogeneity was assessed with Cochran’s Q and I^2^ statistics [33]. The higher the I^2^, the greater the impact of the variance in true effects beyond that depending on sampling error [34].

As in past meta-analyses of single proportions [35, 36], publication bias was not evaluated. Indeed, such kinds of studies are noncomparative; thus, it is unlikely that specific study characteristics may have biased the publications. We used meta-regression techniques to evaluate the impact of the following clinical variables: gender ratio, mean age of the sample, overall sample size, duration of follow-up, tool used for diagnosis. We also investigated the impact of FHR of severe mental illness, as previously delineated, on the risk of transition to psychosis from a CHR-P state.

## Results

### Familial high-risk for severe mental illness

*From the initial set of 2107 records, the literature review and screening resulted in 93 independent samples recruiting CHR-P individuals and assessing the longitudinal outcome (transition vs. no transition). We examined in these studies the habits in reporting familiarity for psychiatric illness (Table *1).

**Table 1:** Studies on CHR-P individuals reporting data on familial high-risk for mental illness

Study, First Author	Year	Nation	Baseline CHR-*P* sample *N*=	Follow up Months	Follow up sample *N*=	CHR-*P* Instrument	Mean age Years (SD)	Gender F %	Broad FHR %	First degree relative with psychosis %	First and/or second degree relative with psychosis %
Yung	2004	Australia	104	12	98	CAARMS	19,4 (3,5)	51		37.5	
Borgwardt	2007	Switzerland	35	12	35	BSIP	23,7 (5,6)	37		14	
Morrison	2007	UK	58	36	27	PANNS	20,96 (5)	31	8.6		
Cannon	2008	USA	370	30	291	SIPS	18,1 (4,6)	41,6	42.6	25.3	16.7
Lemos-Giraldez	2009	Spain	61	36	45	SIPS	21,7 (3,83)	34,4	34.4		
Amminger	2010	Austria	40	12	40	PANSS	16 (1,7)	67,5	25		
Nelson	2011	Australia	817	6	817	CAARMS	18,9 (3.4)	54,3		25.5	
Ziermans	2011	Netherlands	72	12	58	SIPS	15,3 (1)	38,9			16.7
Kim	2012	Korea	78	36	67	CAARMS	21,4 (4,3)	35		16.6	
Koutsouleris	2012	Germany	48	44	35	PANNS	24,7 (0,2)	33,3		35.7	
Schlosser	2012	USA	125	12	84	SIPS	16,9 (3,5)	38		19	
Simon	2012	Switzerland	73	24	42	SIPS	20,4 (5,2)	39,7	16.6		
Lee	2013	Singapore	173	6	173	CAARMS	21,3 (3,5)	32,4	52		58.9
Nieman	2013	Europe	244	18	183	SIPS	22,5 (2,3)	44		16.4	
Katsura	2014	Japan	106	30	83	CAARMS	20 (4,3)	62,3		14.2	
Lindgren	2014	Finland	54	12	51	SIPS	16,7 (0,9)	74,1	24.1	13	11.1
Schultze-Lutter	2014	Germany	194	24	194	SIPS	24,9 (6)	37	9.7		
Zhang	2014	China	117	24	86	SIPS	24,7 (7.6)	52,1		28.1	
Armando	2015	Italy	35	12	35	SIPS	13,8 (2,1)	48,57			11.4
Atkinson	2017	Australia	102	12	68	CAARMS	18,6 (2,7)	40,3		23.5	
Brucato	2017	USA	200	24	200	SIPS	20 (3,9)	27	20.3		17.2
Kotlicka-Antczak	2017	Poland	81	62	65	CAARMS	18,7 (3,5)	51,9		38.2	
Li	2018	China	100	24	91	SIPS	21 (5,4)	57	8	4	
Poletti	2018	Italy	51	24	21	CAARMS	15,4 (1,6)	58,8	60.1	15.7	
Bang	2019	Korea	77	24	77	SIPS	19,9 (3,4)	40,3		15.6	
Chan	2019	Singapore	255	24	255	CAARMS	20,8 (3,3)	32,2	37.9		
Matsumoto	2019	Japan	309	60	307	CAARMS/SIPS	21,4 (5,5)	61,5		14.3	
Yoviene-Sykes	2019	USA	764	12	431	SIPS	19,1 (4,4)	41,8	16.1		
Zarogianni	2019	Switzerland	37	48	35	BSIP	25,2 (6,3)	40	11.4	8.6	2.8
Zhang	2019	China	417	60	349	SIPS	20,9 (6,4)	52	19.6	9.3	
Glenthoj	2020	Denmark	146	12	91	CAARMS	23,9 (4,2)	58,2		21.9	
Pozza	2021	Italy	29	14	19	CAARMS	26 (6)	31		17.2	
Koutsouleris	2021	Europe	167	18	167	SIPS	23,9 (5,4)	49,7		8.4	
Kristensen	2021	Denmark	110	12	88	CAARMS	24 (4)	52,7		23.9	
Mensi	2021	Italy	110	60	103	CAARMS	15,5	62,7	6.1	2.7	3.7
Preti	2021	Italy	125	24	114	BPRS	22 (3)	30	54		
Smesny	2022	Germany	69	12	69	CAARMS	26,2 (6,2)	40,6	20.3		
Pelizza	2023	Italy	178	12	178	CAARMS	19,5 (3,6)	49,5	57.3		

*BPRS* : Brief Psychiatric Rating Scale; *BSIP:* Basel Screening Instrument for Psychosis; *CAARMS:* Comprehensive Assessment of At Risk Mental State; *CHR-P:* Clinical High-Risk for Psychosis; *FHR:* Familial High-Risk; *PANNS:* Positive and Negative Syndrome Scale; *SD:* Standard Deviation; *SIPS:* Structured Interview for Prodromal Symptoms

Broad FHR for severe mental illness was reported in 19 studies out of 93 (20%); information on first-degree relatives with psychosis was reported in 24 studies (26%); information on first- or second-degree relative with a psychosis was reported in 8 studies (9%). When grouped according to the tool used for diagnosis, information about broad FHR for severe mental illness, first-degree relatives with psychosis, and first- or second-degree relative with a psychosis was reported, respectively, in 6 (17%), 11 (31%), and 2 (6%) studies using the CAARMS [37], 9 (19%), 10 (21%), 5 (10%) in studies using the SIPS [38], and 4 (44%), 3 (33%), 1 (11%) in studies using other tools for diagnosing the CHR-P status.

Estimations of proportion of a FHR for severe mental illness at the meta-analytic fixed-effects models were *(See* *Table* 2):

**Table 2:** Main results on familiarity variables

Familiarity features	% of studies reporting data	Meta-analytical prevalence
Broad family history of psychiatric illness	20%	29.9%
Family history of first-degree relatives with psychosis	26%	21.0%
Family history of first- or second-degree relative with a psychosis	9%	24.6%

- for broad family history of psychiatric illness, 29.9% (95%CI: 28.2% − 31.6%); Cochran’s Q = 376.2; df = 18; *p* < 0.0001; I^2^ = 95% (93% − 96%) (see Figure S2n Supplementary Materials for details);
- for family history of first-degree relatives with psychosis, 21.0% (19.7% − 22.4%); Cochran’s Q = 152.7; df = 23; *p* < 0.0001; I^2^ = 85% (79% − 89%) (see Figure S3in Supplementary Materials for details);
- for family history of first- or second-degree relative with a psychosis, 24.6% (21.7% − 27.7%); Cochran’s Q = 143.9; df = 7; *p* < 0.0001; I^2^ = 95% (92% − 97%) (see Figure S4 In Supplementary Materials for details).

Heterogeneity was substantial in all estimations (I^2^ > 75%).

Estimates of broad FHR for severe mental illness were not influenced by gender proportion (beta=−0.009; s.e.=0.017; t=−0.528; *p* = 0.604), age (beta=−0.063; s.e.=0.076; t=−0.818; *p* = 0.424), sample size (beta=−0.001; s.e.=0.001; t=−0.025; *p* = 0.980). As for the tool used for diagnosis, studies using SIPS reported a lower proportion of broad FHR for severe mental illness (23%; 21%−25%) than those using CAARMS (44%; 40%−48%) or other tools (38%; 32%−46%): Q = 122.3; df = 2; *p* < 0.0001.

Estimates of family history of first-degree relatives with psychosis were not influenced by gender proportion (beta=−0.017; s.e.=0.014; t=−1.276; *p* = 0.215), age (beta = 0.018; s.e.=0.050; t = 0.364; *p* = 0.719), sample size (beta=−0.001; s.e.=0.001; t = 0.300; *p* = 0.767). As for the tool used for diagnosis, again studies using SIPS reported a lower proportion of family history of first-degree relatives with psychosis (17%; 15%−19%) than those using CAARMS (25%; 23%−27%) or other tools (24%; 17%−33%): Q = 30.9; df = 2; *p* < 0.0001.

Estimates of family history of first- or second-degree relative with a psychosis were not influenced by gender proportion (beta=−0.037; s.e.=0.025; t=−1.460; *p* = 0.194), age (beta = 0.055; s.e.=0.137; t = 0.399; *p* = 0.703), sample size (beta = 0.003; s.e.=0.004; t = 0.924; *p* = 0.391). As for the tool used for diagnosis, the studies using CAARMS reported a higher proportion of family history of first- or second-degree relative with a psychosis (51%; 44%−58%) than those using SIPS (16%; 14%−19%) or other tools for diagnosis (3%; 0.4%−17%): Q = 95.2; df = 2; *p* < 0.0001.

### Transition to psychosis from a CHR-P state

Transition to psychosis across the reviewed 93 studies was 18.6% (17.8% − 19.3%) at the fixed-effects model and 18.7% (16.6% − 20.9%) at the random-effects model. Heterogeneity was substantial: Cochran’s Q = 554.7; df = 92; *p* < 0.0001; I^2^ = 83% (80% − 86%).

**Fig. 1:**
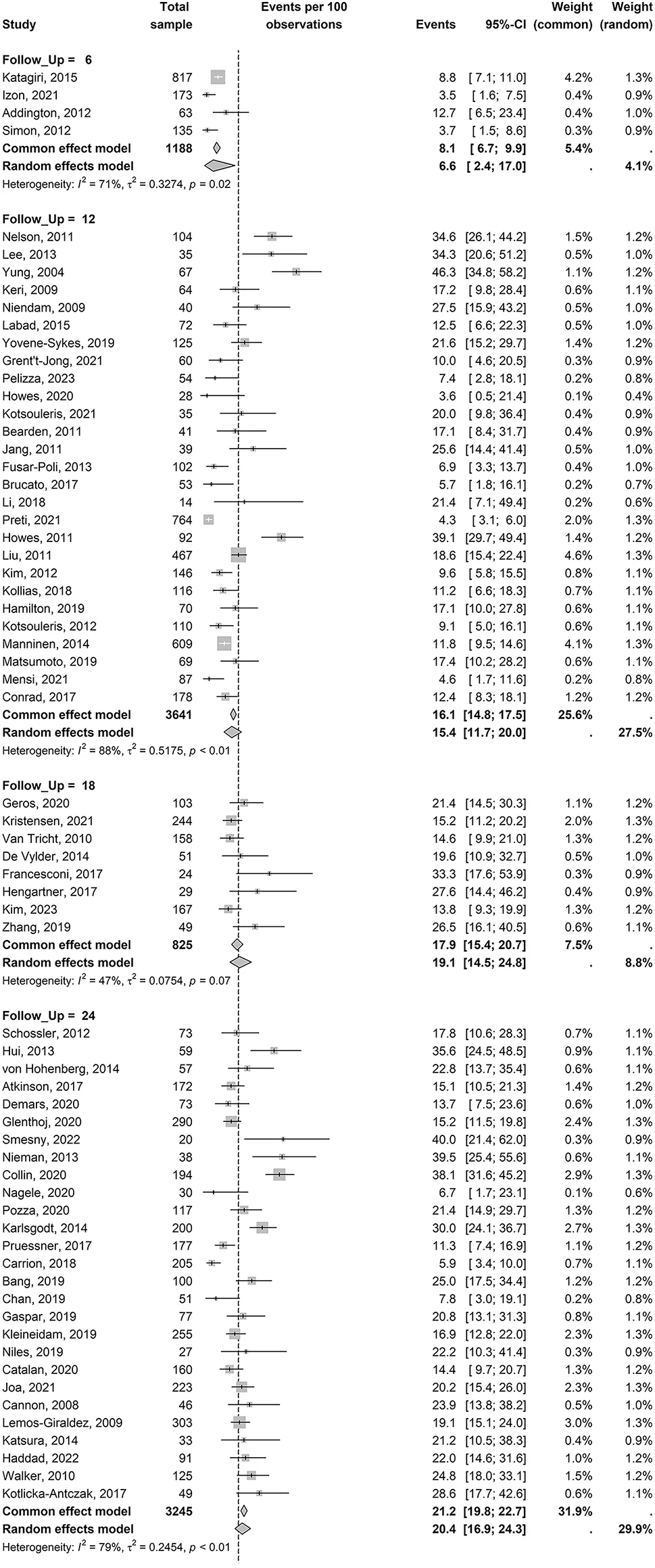

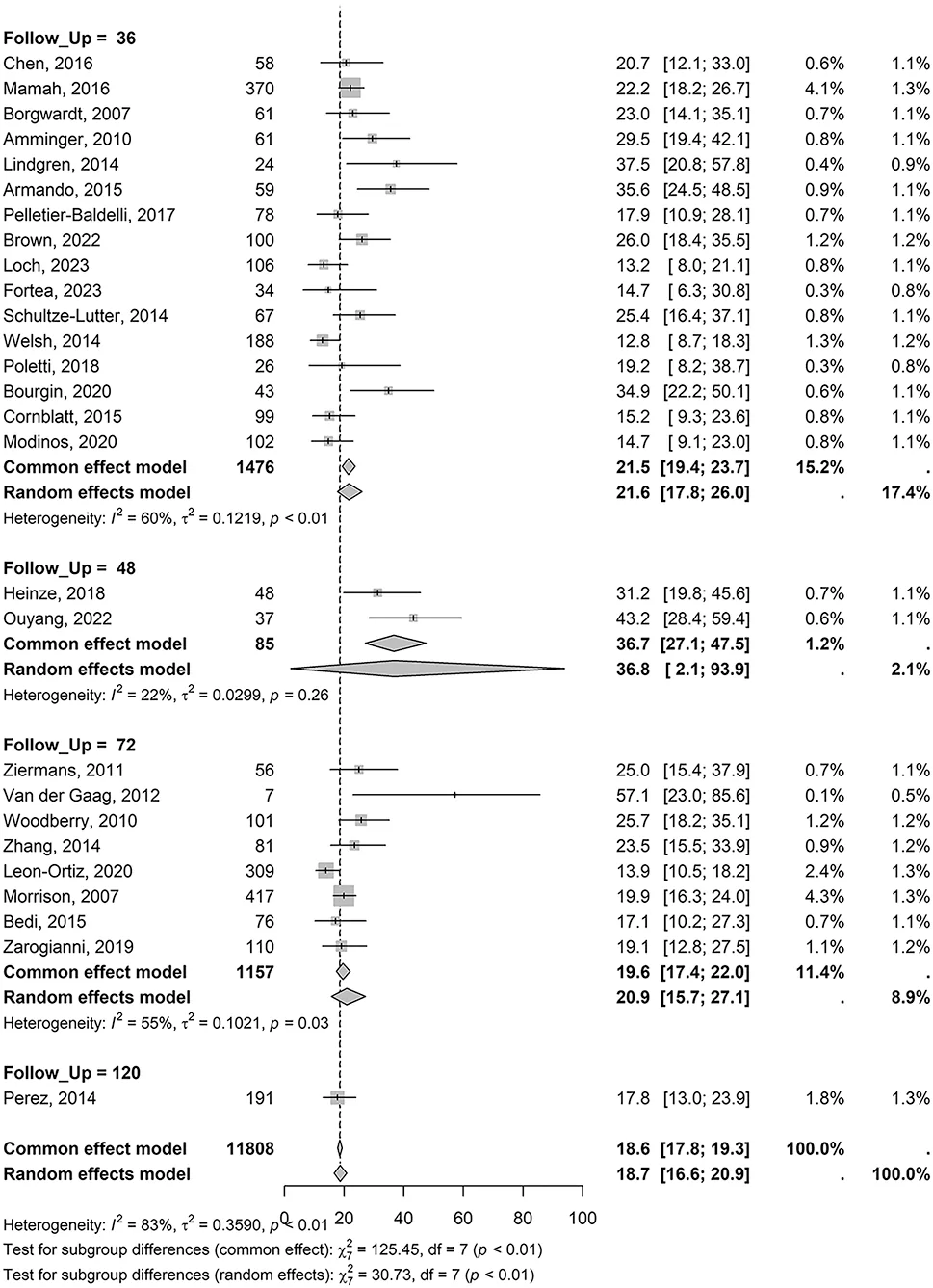
Forest plot of transition rates to psychosis by length of follow-up among individuals at clinical high risk for psychosis

An increase of transition risk across time is evident from 6 months (fixed-effects model estimates: 8.1% [6.6% − 9.9%]; random-effects model estimates: 6.6% [2.3% − 16.9%]) up to 48 months (fixed-effects model estimates: 36.6% [27.0% − 47.5%]; random-effects model estimates: 36.7% [2.1% − 93.9%]) and decays thereafter (Fig. 1).

The effect of follow-up upon transition risk was evident at both the fixed-effects model (between groups Q = 125.4; df = 7; *p* < 0.0001) and the random-effects model (between groups Q = 30.7; df = 7; *p* < 0.0001). However, the amount of heterogeneity accounted for because of follow-up was modest: 6.2%.

**Fig. 2:**
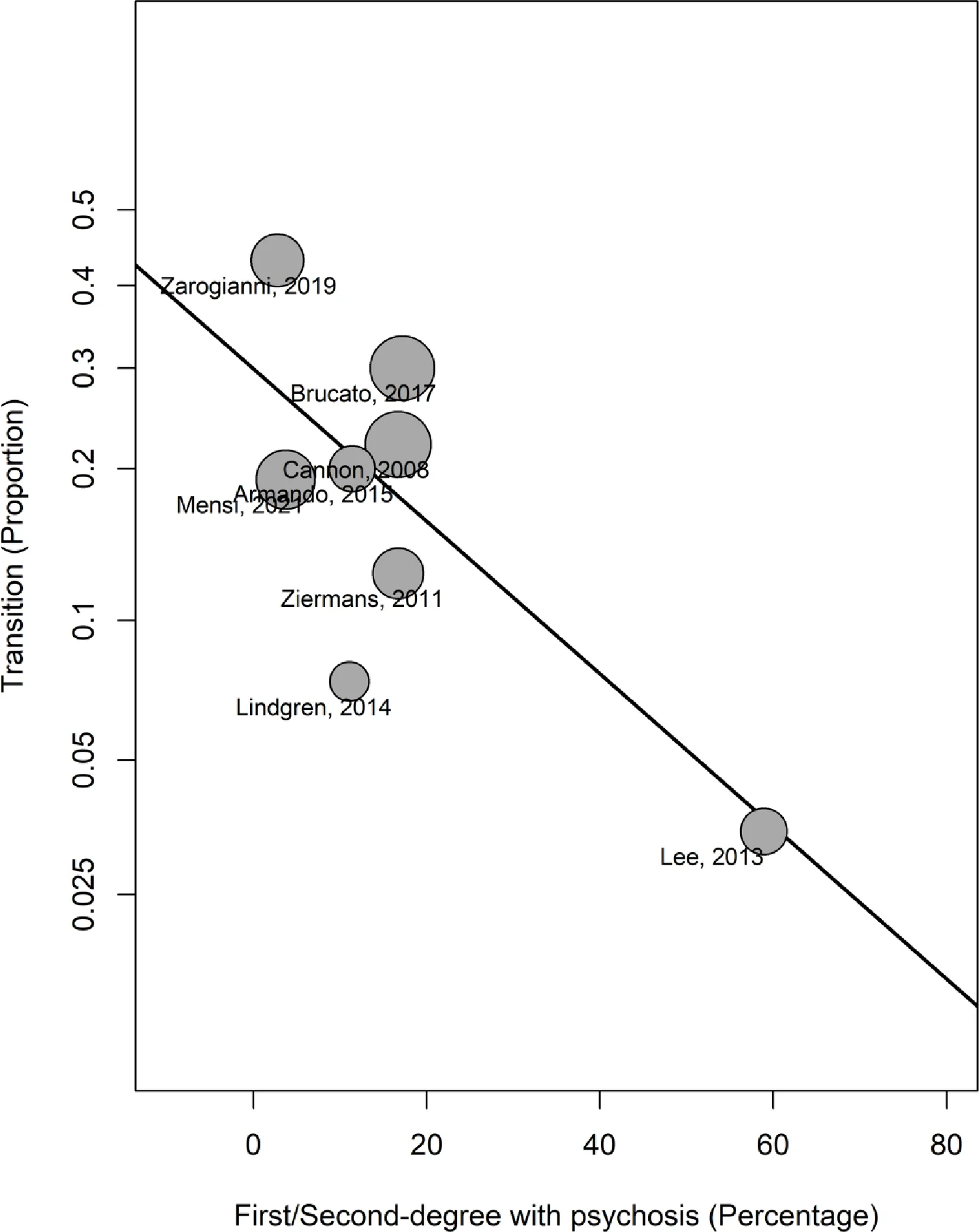
Bubble plot showing the relationship between the proportion of individuals at clinical high risk for psychosis who transitioned to psychosis (vertical axis) and the percentage with a first- or second-degree family history of psychosis (horizontal axis)

Family history of first- or second-degree relative with a psychosis was negatively related to the transition risk in the subgroup of studies reporting the information (k = 8): beta=−0.041; s.e.=0.015; t=−2.722; *p* = 0.034; heterogeneity accounted for = 56.6% (Fig. 2).

Broad FHR for severe mental illness (k = 17) was not related to transition risk (beta=−0.017; s.e.=0.012; t=−1.454; *p* = 0.166), neither it was a family history of first-degree relatives with psychosis (k = 24; beta = 0.005; s.e.=0.013; t = 0.374; *p* = 0.712).

## Discussion

This systematic review and meta-analysis had three main objectives regarding FHR for severe mental illness in observational cohorts CHR-P individuals *following the longitudinal risk of transition to psychosis*:

- to map reporting habits;
- to quantify the prevalence of different FHR definitions;
- to determine its possible impact on the transition to psychosis.

### Reporting habits

The systematic review found that only a minority of included CHR-P studies reported any information related to FHR for psychosis. Approximately 20% of the 93 included studies provided data on broad FHR for severe mental illness, 26% reported information on first-degree relatives with psychosis, and 9% reported on first- or second-degree relatives with psychosis. This pattern suggests that FHR information is undervalued and not systematically reported in CHR-P studies, despite its importance in properly classifying CHR-P subgroups, particularly GRFD. The lack of detailed FHR data is a significant obstacle to providing accurate prognostic estimates.

We were able to retrieve information on family history of mental disorders - especially within the psychosis spectrum - in only a minority of the reviewed studies. The lack of transparency, or even widespread omission, of such a central clinical-anamnestic feature represents a serious limitation in the field [6]. This gap may impair the accuracy of predictive models and ultimately hinder the development of truly personalized, tailored interventions.* It must be underlined as possible limitation that the focus on CHR-P studies longitudinally the yes/no transition outcome may have partially influenced the mapping of the whole CHR-P literature, and other cross-sectional studies on CHR-P may have reported data on FHR variables. Also the choice of databases for literature review could have influenced results. Our search included PubMed and the Cochrane Library. Although extending the search to additional databases such as Embase or PsycINFO might have identified some further European or psychology-focused studies, evidence suggests that the incremental yield beyond MEDLINE is generally modest [*39, 40*]. Most studied datasets on CHR-P were included and the number of included studies (n = 93) is one of the highest among meta-analyses on CHR-P cohorts, therefore we deem that our meta-analytical estimation is quite representative of the CHR-P field.*

### Prevalence of FHR

The prevalence estimates of FHR for severe mental illness in observational CHR-P cohorts exhibited substantial heterogeneity. However, the available data were consistent with previous findings in related research areas. Specifically, the estimated prevalence of broad FHR for severe mental illness (30%; 28%–32%) was close to the 27.1% found in a large Danish sample of 9,324 individuals diagnosed with schizophrenia [41]. It was also lower than the 39.5% reported in a Danish population study on bipolar affective disorder [42]. Furthermore, this prevalence aligns with the meta-analytically derived 32% probability of developing a severe mental illness in offspring of parents with schizophrenia or affective disorders [15].

Importantly, heterogeneity in this study was not influenced by sample composition (e.g., size, mean age, or gender proportion). Instead, the primary source of heterogeneity appeared to be the method of sample enrollment, particularly the diagnostic tool used. Studies employing the CAARMS tended to place greater emphasis on family history of psychiatric illness, especially psychosis in first- and second-degree relatives. This is reflected in the higher proportion of GRFD cases detected in studies using the CAARMS compared to those using the SIPS, another widely used tool for diagnosing CHR-P status. However, even among CAARMS-based studies, family history of mental disorders was reported in only a minority of cases - data on psychosis in first-degree relatives were provided in just 33% of the reviewed studies. Studies using other diagnostic tools, such as the SIPS, were no more likely to report family history information.

### Impact of FHR on transition to psychosis

Regarding the potential impact of FHR for severe mental illness on longitudinal outcomes, we found that a family history of psychosis in first- or second-degree relatives was negatively associated with transition risk in the subgroup of studies (*n* = 8) that reported this information. In contrast, broad FHR for severe mental illness (*n* = 19) and family history of psychosis in first-degree relatives (*n* = 24) were not significantly related to transition risk.

The unexpected finding of a negative association requires careful interpretation, given several methodological considerations. First, the small number of studies reporting FHR data, along with their heterogeneity and the statistical methods used (i.e., meta-regressions based on percentages rather than individual raw data), limits the robustness of this result. Consequently, greater reliance should be placed on findings from the larger subsets of studies (19 and 24 studies) that suggest no clear association rather than on the negative relation observed in only 8 studies.

Crucially, the absence of evidence for an FHR effect on transition to psychosis should not be interpreted as a disconfirmation of the clinical relevance of heritability and familial risk. The role of FHR should be further investigated using larger datasets and individual-level data (e.g., transition yes/no in CHR-P individuals with and without FHR), rather than aggregated percentages. Additionally, it is important to consider that:

- Unlike well-established severe mental illnesses such as schizophrenia and affective disorders, CHR-P criteria capture a broader help-seeking population at presumed prodromal stages of psychosis.
- Psychometric transition to psychosis - based on the same scales used to assess CHR-P - is only one of several possible psychopathological outcomes [43]. Various confounders, such as exposure to antipsychotics [4, 7–9], may influence the longitudinal relationship between FHR and clinical outcomes.

## Conclusions

This study found that, despite the importance of assessing familial risk for severe mental illness in help-seeking individuals, only a minority of CHR-P studies focusing on transition rates as outcome report such information. Although operational definitions of FHR varied from broad to narrow, the estimated prevalence of FHR in CHR-P individuals was similar to that observed in schizophrenia-spectrum disorders and slightly lower than in bipolar disorder. Contrary to expectations, FHR did not appear to increase the immediate risk of transition to psychosis. However, this finding requires further meta-analytical replication using larger datasets with individual-level data. Given the current limitations - particularly the small number of studies and statistical constraints imposed by available data (meta-regressions based on percentages) - these results should be interpreted with caution.

## Supplementary Information

Below is the link to the electronic supplementary material.

Supplementary Material 1

## Data Availability

All used data are published in the paper.
