# Supplementary material for: Familial high-risk (FHR) for severe mental illness in clinical high-risk for psychosis (CHR-P): a systematic review and meta-analysis
Source: Eur Arch Psychiatry Clin Neurosci. 2026 May 28;276(6):3077–86. doi: 10.1007/s00406-026-02267-1 (PMC13661641; doi:10.1007/s00406-026-02267-1)
Supplement: Supplementary file 1 [file 406_2026_2267_MOESM1_ESM.docx]

**Familial high-risk (FHR) for severe mental illness in Clinical High-Risk for Psychosis (CHR-P): an epidemiological systematic review and meta-analysis of reporting habits and prevalence**

**Supplementary materials**

**Results**

*Samples’ characteristics*

The sample size at baseline of the reviewed studies included an average of 127 participants (standard deviation [SD]: 142); 40% of the studies had a baseline sample size of 100 participants or higher and just 25% of the studies had a sample size with less than 50 participants. The studies included samples with an age range between 13.8 and 26, with an average of 20 years old (SD: 3). On the basis of mean age and standard deviation as reported in the studies, we classified them in 18 studies (19%) including only children and adolescents (age<18), 14 studies (15%) including mixed samples of both children and adolescents and adults (mean age: 17 to 18 with SD around 3), and 61 (66%) studies with samples of adults only (mean age≥19). Proportion of females ranged from 15% to 76%, averaging 44%. The tool used to diagnose the CHR-P status and define the outcome of transition to psychosis was the Comprehensive Assessment of at-risk Mental States (CAARMS) [1] in 36 studies (39%), the Structured Interview of Psychosis-risk Syndromes (SIPS) [2] in 47 studies (51%), one study used both CAARMS and SIPS but resorted to SIPS for transition, and 9 studies (9%) used other tools, such as the Positive and Negative Symptoms Scale (PANSS) [3] in 6 studies, the Basel Screening Instrument for Psychosis (BSIP) [4] in 2 studies, and the Brief Psychiatric Rating Scale (BPRS) [5] in 1 study. In these studies, the follow-up varied from 6 to 120 months, with 26 months, on average, of follow-up.

*References for assessment scales*

1. Yung AR, Yuen HP, McGorry PD, et al (2005) Mapping the onset of psychosis: the Comprehensive Assessment of At-Risk Mental States. Aust N Z J Psychiatry 39:964-971. doi:10.1080/j.1440-1614.2005.01714.x
2. McGlashan TH. Structured Interview for Prodromal Symptoms (SIPS). Yale University, 2001.
3. Kay SR, Fiszbein A, Opler LA (1987) The positive and negative syndrome scale (PANSS) for schizophrenia. Schizophr Bull 13:261-276. doi:10.1093/schbul/13.2.261
4. Riecher-Rössler A, Aston J, Ventura J et al (2008) Das Basel Screening Instrument für Psychosen (BSIP): Entwicklung, Aufbau, Reliabilität und Validität [The Basel Screening Instrument for Psychosis (BSIP): development, structure, reliability and validity]. Fortschritte der Neurologie-Psychiatrie, 76:207–216. https://doi.org/10.1055/s-2008-1038155
5. Overall JE, Gorham DR (1962) The Brief Psychiatric Rating Scale. Psychol Rep 10:799-812. doi:10.2466/pr0.1962.10.3.799

**Figure S1.** PRISMA flowchart of included studies reporting familial high-risk for severe mental illness in individuals at clinical high risk (CHR) for psychosis.

**Identification of studies via databases and registers**

Records removed *before screening*:

Duplicate records removed (n = 5)

Records marked as ineligible by automation tools (n = 15)

Records removed for other reasons, i.e. systematic reviews or meta-analysis (n = 176)

Records identified from*:

Databases (n = 2107)

Registers (n = 0)

**Identification**

Records screened

(n = 1911)

Records excluded**

(n = 1597)

Reports sought for retrieval

(n = 314)

Reports not retrieved

(n = 0)

**Screening**

Reports excluded:

No CHR-P assessment (n = 2)

Overlapping samples (n =219)

Reports assessed for eligibility

(n = 314)

Studies included in review

(n = 93)

Reports of included studies

(n = 93)

**Included**

*Consider, if feasible to do so, reporting the number of records identified from each database or register searched (rather than the total number across all databases/registers).

**If automation tools were used, indicate how many records were excluded by a human and how many were excluded by automation tools.

Source: Page MJ, et al. BMJ 2021;372:n71. doi: 10.1136/bmj.n71.This work is licensed under CC BY 4.0. To view a copy of this license, visit <https://creativecommons.org/licenses/by/4.0/>

**Figure S2**. Forest plot showing the proportion of individuals at clinical high risk for psychosis with a broad family history of psychiatric illness.

**Figure S3**. Forest plot showing the proportion of individuals at clinical high risk for psychosis with a first-degree family history of psychosis.

**Figure S4**. Forest plot showing the proportion of individuals at clinical high risk for psychosis with a first- or second-degree family history of psychosis.

**References for studies reporting data on familiarity**

- Amminger, G. P., Schäfer, M. R., Papageorgiou, K., Klier, C. M., Cotton, S. M., Harrigan, S. M., Mackinnon, A., McGorry, P. D., & Berger, G. E. (2010). Long-chain omega-3 fatty acids for indicated prevention of psychotic disorders: a randomized, placebo-controlled trial. *Archives of General Psychiatry, 67*(2), 146–154. <https://doi.org/10.1001/archgenpsychiatry.2009.192>
- Armando, M., Pontillo, M., De Crescenzo, F., Mazzone, L., Monducci, E., Lo Cascio, N., Santonastaso, O., Pucciarini, M. L., Vicari, S., Schimmelmann, B. G., & Schultze-Lutter, F. (2015). Twelve-month psychosis-predictive value of the ultra-high risk criteria in children and adolescents. *Schizophrenia Research, 169*(1-3), 186–192. <https://doi.org/10.1016/j.schres.2015.10.033>
- Atkinson, R. J., Fulham, W. R., Michie, P. T., Ward, P. B., Todd, J., Stain, H., Langdon, R., Thienel, R., Paulik, G., Cooper, G., MinT Consortium, & Schall, U. (2017). Electrophysiological, cognitive and clinical profiles of at-risk mental state: The longitudinal Minds in Transition (MinT) study. *PloS one, 12*(2), e0171657. <https://doi.org/10.1371/journal.pone.0171657>
- Bang, M., Park, J. Y., Kim, K. R., Lee, S. Y., Song, Y. Y., Kang, J. I., Lee, E., & An, S. K. (2019). Psychotic conversion of individuals at ultra-high risk for psychosis: The potential roles of schizotypy and basic symptoms. *Early Intervention in Psychiatry, 13*(3), 546–554. <https://doi.org/10.1111/eip.12518>
- Borgwardt, S. J., McGuire, P. K., Aston, J., Berger, G., Dazzan, P., Gschwandtner, U., Pflüger, M., D'Souza, M., Radue, E. W., & Riecher-Rössler, A. (2007). Structural brain abnormalities in individuals with an at-risk mental state who later develop psychosis. *British Journal of Psychiatry. Supplement, 51*, s69–s75. <https://doi.org/10.1192/bjp.191.51.s69>
- Brucato, G., Masucci, M. D., Arndt, L. Y., Ben-David, S., Colibazzi, T., Corcoran, C. M., Crumbley, A. H., Crump, F. M., Gill, K. E., Kimhy, D., Lister, A., Schobel, S. A., Yang, L. H., Lieberman, J. A., & Girgis, R. R. (2017). Baseline demographics, clinical features and predictors of conversion among 200 individuals in a longitudinal prospective psychosis-risk cohort. *Psychological Medicine, 47*(11), 1923–1935. <https://doi.org/10.1017/S0033291717000319>
- Cannon, T. D., Cadenhead, K., Cornblatt, B., Woods, S. W., Addington, J., Walker, E., Seidman, L. J., Perkins, D., Tsuang, M., McGlashan, T., & Heinssen, R. (2008). Prediction of psychosis in youth at high clinical risk: a multisite longitudinal study in North America. *Archives of General Psychiatry, 65*(1), 28–37. <https://doi.org/10.1001/archgenpsychiatry.2007.3>
- Chan, C. T., Abdin, E., Subramaniam, M., Tay, S. A., Lim, L. K., & Verma, S. (2019). Two-Year Clinical and Functional Outcomes of an Asian Cohort at Ultra-High Risk of Psychosis. *Frontiers in Psychiatry, 9*, 758. <https://doi.org/10.3389/fpsyt.2018.00758>
- Glenthøj, L. B., Kristensen, T. D., Wenneberg, C., Hjorthøj, C., & Nordentoft, M. (2021). Predictors of remission from the ultra-high risk state for psychosis. *Early Intervention in Psychiatry, 15*(1), 104–112. <https://doi.org/10.1111/eip.12917>
- Izon, E., Berry, K., Wearden, A., Carter, L. A., Law, H., & French, P. (2021). Investigating expressed emotion in individuals at-risk of developing psychosis and their families over 12 months. *Clinical Psychology & Psychotherapy, 28*(5), 1285–1296. <https://doi.org/10.1002/cpp.2576>
- Katsura, M., Ohmuro, N., Obara, C., Kikuchi, T., Ito, F., Miyakoshi, T., Matsuoka, H., & Matsumoto, K. (2014). A naturalistic longitudinal study of at-risk mental state with a 2.4 year follow-up at a specialized clinic setting in Japan. *Schizophrenia Research, 158*(1-3), 32–38. https://doi.org/10.1016/j.schres.2014.06.013
- Kim, E., Jang, J. H., Park, H. Y., Shim, G., Hwang, J. Y., Kim, S. N., & Kwon, J. S. (2012). Pharmacotherapy and clinical characteristics of ultra-high-risk for psychosis according to conversion status: a naturalistic observational study. *Early Intervention in Psychiatry, 6*(1), 30–37. <https://doi.org/10.1111/j.1751-7893.2011.00295.x>
- Kotlicka-Antczak, M., Pawełczyk, A., Karbownik, M. S., Pawełczyk, T., Strzelecki, D., Żurner, N., & Urban-Kowalczyk, M. (2017). Deficits in the identification of pleasant odors predict the transition of an at-risk mental state to psychosis. *Schizophrenia Research, 181*, 49–54. <https://doi.org/10.1016/j.schres.2016.10.019>
- Koutsouleris, N., Davatzikos, C., Bottlender, R., Patschurek-Kliche, K., Scheuerecker, J., Decker, P., Gaser, C., Möller, H. J., & Meisenzahl, E. M. (2012). Early recognition and disease prediction in the at-risk mental states for psychosis using neurocognitive pattern classification. *Schizophrenia Bulletin, 38*(6), 1200–1215. <https://doi.org/10.1093/schbul/sbr037>
- Koutsouleris, N., Dwyer, D. B., Degenhardt, F., Maj, C., Urquijo-Castro, M. F., Sanfelici, R., Popovic, D., Oeztuerk, O., Haas, S. S., Weiske, J., Ruef, A., Kambeitz-Ilankovic, L., Antonucci, L. A., Neufang, S., Schmidt-Kraepelin, C., Ruhrmann, S., Penzel, N., Kambeitz, J., Haidl, T. K., Rosen, M., … PRONIA Consortium (2021). Multimodal Machine Learning Workflows for Prediction of Psychosis in Patients With Clinical High-Risk Syndromes and Recent-Onset Depression. *JAMA Psychiatry, 78*(2), 195–209. <https://doi.org/10.1001/jamapsychiatry.2020.3604>
- Kristensen, T. D., Glenthøj, L. B., Ambrosen, K., Syeda, W., Raghava, J. M., Krakauer, K., Wenneberg, C., Fagerlund, B., Pantelis, C., Glenthøj, B. Y., Nordentoft, M., & Ebdrup, B. H. (2021). Global fractional anisotropy predicts transition to psychosis after 12 months in individuals at ultra-high risk for psychosis. *Acta Psychiatrica Scandinavica, 144*(5), 448–463. <https://doi.org/10.1111/acps.13355>
- Lee, J., Rekhi, G., Mitter, N., Bong, Y. L., Kraus, M. S., Lam, M., Rapisarda, A., Lee, T. S., Subramaniam, M., Chong, S. A., & Keefe, R. S. (2013). The Longitudinal Youth at Risk Study (LYRIKS)--an Asian UHR perspective. *Schizophrenia Research, 151*(1-3), 279–283. <https://doi.org/10.1016/j.schres.2013.09.025>
- Lemos-Giráldez, S., Vallina-Fernández, O., Fernández-Iglesias, P., Vallejo-Seco, G., Fonseca-Pedrero, E., Paíno-Piñeiro, M., Sierra-Baigrie, S., García-Pelayo, P., Pedrejón-Molino, C., Alonso-Bada, S., Gutiérrez-Pérez, A., & Ortega-Ferrández, J. A. (2009). Symptomatic and functional outcome in youth at ultra-high risk for psychosis: a longitudinal study. *Schizophrenia Research, 115*(2-3), 121–129. <https://doi.org/10.1016/j.schres.2009.09.011>
- Li, H., Zhang, T., Xu, L., Tang, Y., Cui, H., Wei, Y., Tang, X., Woodberry, K. A., Shapiro, D. I., Li, C., Seidman, L. J., & Wang, J. (2018). A comparison of conversion rates, clinical profiles and predictors of outcomes in two independent samples of individuals at clinical high risk for psychosis in China*. Schizophrenia Research, 197*, 509–515. <https://doi.org/10.1016/j.schres.2017.11.029>
- Lindgren, M., Manninen, M., Kalska, H., Mustonen, U., Laajasalo, T., Moilanen, K., Huttunen, M., Cannon, T. D., Suvisaari, J., & Therman, S. (2014). Predicting psychosis in a general adolescent psychiatric sample. *Schizophrenia Research, 158*(1-3), 1–6. <https://doi.org/10.1016/j.schres.2014.06.028>
- Matsumoto, K., Katsura, M., Tsujino, N., Nishiyama, S., Nemoto, T., Katagiri, N., Takahashi, T., Higuchi, Y., Ohmuro, N., Matsuoka, H., Suzuki, M., & Mizuno, M. (2019). Federated multi-site longitudinal study of at-risk mental state for psychosis in Japan. *Schizophrenia Research, 204*, 343–352. <https://doi.org/10.1016/j.schres.2018.09.001>
- Mensi, M. M., Molteni, S., Iorio, M., Filosi, E., Ballante, E., Balottin, U., Fusar-Poli, P., & Borgatti, R. (2021). Prognostic Accuracy of DSM-5 Attenuated Psychosis Syndrome in Adolescents: Prospective Real-World 5-Year Cohort Study. *Schizophrenia Bulletin, 47*(6), 1663–1673. <https://doi.org/10.1093/schbul/sbab041>
- Morrison, A. P., French, P., Parker, S., Roberts, M., Stevens, H., Bentall, R. P., & Lewis, S. W. (2007). Three-year follow-up of a randomized controlled trial of cognitive therapy for the prevention of psychosis in people at ultrahigh risk. *Schizophrenia Bulletin, 33*(3), 682–687. <https://doi.org/10.1093/schbul/sbl042>
- Nelson, B., Yuen, K., & Yung, A. R. (2011). Ultra high risk (UHR) for psychosis criteria: are there different levels of risk for transition to psychosis?. *Schizophrenia Research, 125*(1), 62–68. <https://doi.org/10.1016/j.schres.2010.10.017>
- Nieman, D. H., Velthorst, E., Becker, H. E., de Haan, L., Dingemans, P. M., Linszen, D. H., Birchwood, M., Patterson, P., Salokangas, R. K., Heinimaa, M., Heinz, A., Juckel, G., von Reventlow, H. G., Morrison, A., Schultze-Lutter, F., Klosterkötter, J., Ruhrmann, S., & EPOS group (2013). The Strauss and Carpenter Prognostic Scale in subjects clinically at high risk of psychosis. *Acta Psychiatrica Scandinavica, 127*(1), 53–61. <https://doi.org/10.1111/j.1600-0447.2012.01899.x>
- Pelizza, L., Leuci, E., Quattrone, E., Azzali, S., Paulillo, G., Pupo, S., Poletti, M., Raballo, A., Pellegrini, P., & Menchetti, M. (2024). Baseline antipsychotic prescription and short-term outcome indicators in individuals at clinical high-risk for psychosis: Findings from the Parma At-Risk Mental States (PARMS) program. *Early Intervention in Psychiatry, 18*(2), 71–81. <https://doi.org/10.1111/eip.13434>
- Poletti, M., Pelizza, L., Azzali, S., Paterlini, F., Garlassi, S., Scazza, I., Chiri, L. R., Gebhardt, E., Pupo, S., & Andrea, R. (2019). Clinical high risk for psychosis in childhood and adolescence: findings from the 2-year follow-up of the ReARMS project. *European Child & Adolescent Psychiatry, 28*(7), 957–971. <https://doi.org/10.1007/s00787-018-1262-5>
- Pozza, A., Domenichetti, S., & Dèttore, D. (2021). Cognitive behavioural therapy for help-seeking adolescents and young adults with at-risk-mental state: Effects on subclinical positive symptoms. *Early Intervention in Psychiatry, 15*(3), 513–524. <https://doi.org/10.1111/eip.12974>
- Preti, A., Raballo, A., Meneghelli, A., Cocchi, A., Meliante, M., Barbera, S., Malvini, L., Monzani, E., & Percudani, M. (2022). Antipsychotics are related to psychometric conversion to psychosis in ultra-high-risk youth. Early intervention in psychiatry, 16(4), 342–351. <https://doi.org/10.1111/eip.13158>
- Schlosser, D. A., Jacobson, S., Chen, Q., Sugar, C. A., Niendam, T. A., Li, G., Bearden, C. E., & Cannon, T. D. (2012). Recovery from an at-risk state: clinical and functional outcomes of putatively prodromal youth who do not develop psychosis. *Schizophrenia Bulletin, 38*(6), 1225–1233. <https://doi.org/10.1093/schbul/sbr098>
- Schultze-Lutter, F., Klosterkötter, J., & Ruhrmann, S. (2014). Improving the clinical prediction of psychosis by combining ultra-high risk criteria and cognitive basic symptoms. *Schizophrenia Research, 154*(1-3), 100–106. <https://doi.org/10.1016/j.schres.2014.02.010>
- Simon, A. E., Grädel, M., Cattapan-Ludewig, K., Gruber, K., Ballinari, P., Roth, B., & Umbricht, D. (2012). Cognitive functioning in at-risk mental states for psychosis and 2-year clinical outcome. *Schizophrenia Research, 142*(1-3), 108–115. <https://doi.org/10.1016/j.schres.2012.09.004>
- Smesny, S., Gussew, A., Schack, S., Langbein, K., Wagner, G., & Reichenbach, J. R. (2022). Neurometabolic patterns of an "at risk for mental disorders" syndrome involve abnormalities in the thalamus and anterior midcingulate cortex. *Schizophrenia Research, 243*, 285–295. <https://doi.org/10.1016/j.schres.2020.04.021>
- Yoviene Sykes, L. A., Ferrara, M., Addington, J., Bearden, C. E., Cadenhead, K. S., Cannon, T. D., Cornblatt, B. A., Perkins, D. O., Mathalon, D. H., Seidman, L. J., Tsuang, M. T., Walker, E. F., McGlashan, T. H., Woodberry, K. A., Powers, A. R., 3rd, Ponce, A. N., Cahill, J. D., Pollard, J. M., Srihari, V. H., & Woods, S. W. (2020). Predictive validity of conversion from the clinical high risk syndrome to frank psychosis. *Schizophrenia Research, 216*, 184–191. <https://doi.org/10.1016/j.schres.2019.12.002>
- Yung, A. R., Phillips, L. J., Yuen, H. P., & McGorry, P. D. (2004). Risk factors for psychosis in an ultra high-risk group: psychopathology and clinical features. *Schizophrenia Research, 67*(2-3), 131–142. <https://doi.org/10.1016/S0920-9964(03)00192-0>
- Zarogianni, E., Storkey, A. J., Borgwardt, S., Smieskova, R., Studerus, E., Riecher-Rössler, A., & Lawrie, S. M. (2019). Individualized prediction of psychosis in subjects with an at-risk mental state. *Schizophrenia Research, 214*, 18–23. <https://doi.org/10.1016/j.schres.2017.08.061>
- Zhang, T., Li, H., Woodberry, K. A., Seidman, L. J., Zheng, L., Li, H., Zhao, S., Tang, Y., Guo, Q., Lu, X., Zhuo, K., Qian, Z., Chow, A., Li, C., Jiang, K., Xiao, Z., & Wang, J. (2014). Prodromal psychosis detection in a counseling center population in China: an epidemiological and clinical study. *Schizophrenia Research, 152*(2-3), 391–399. <https://doi.org/10.1016/j.schres.2013.11.039>
- Zhang, T., Xu, L., Tang, Y., Li, H., Tang, X., Cui, H., Wei, Y., Wang, Y., Hu, Q., Liu, X., Li, C., Lu, Z., McCarley, R. W., Seidman, L. J., Wang, J., & SHARP (ShangHai At Risk for Psychosis) Study Group (2019). Prediction of psychosis in prodrome: development and validation of a simple, personalized risk calculator. *Psychological Medicine, 49*(12), 1990–1998. <https://doi.org/10.1017/S0033291718002738>
- Ziermans, T. B., Schothorst, P. F., Sprong, M., & van Engeland, H. (2011). Transition and remission in adolescents at ultra-high risk for psychosis. *Schizophrenia Research, 126*(1-3), 58–64. <https://doi.org/10.1016/j.schres.2010.10.022>
